# Psychological drivers of electric vehicle battery recycling: the impact of place attachment and sustainable attitudes

**DOI:** 10.3389/fpsyg.2025.1634913

**Published:** 2026-01-22

**Authors:** Jie Cheng, Tian Gao, Yi-Cheng Zhang, Noor Ullah Khan, Ke-Bin Lu

**Affiliations:** 1 School of Foreign Languages, Huangshan University, Huangshan, China; 2 School of Earth and Space Sciences, University of Science and Technology of China, Hefei, China; 3 School of Business, Suzhi Education Centre, Anhui Xinhua University, Hefei, China; 4 Faculty of Business and Management, Universiti Teknologi MARA, Cawangan Selangor, Puncak Alam, Malaysia

**Keywords:** place attachment, place identity, social bonding, natural bonding, sustainable attitude, intention to formal recycling

## Abstract

**Introduction:**

The rapid expansion of electric‑vehicle adoption in China has intensified concerns about end‑of‑life management of power batteries. Despite increasing apprehensions about economic, social, and environmental sustainability 2021-2035, there has been a noticeable surge in scholarly interest directed towards formal power battery recycling. Although psychological drivers are key to participation through the lens of behavioral reasoning theory, the role of place attachment remains underexplored, particularly in collectivist cultures. Bridging this research lacuna, the current study offers an in-depth and holistic investigation of how the multidimensional facets of place attachment influence residents’ economic, social, and environmental attitudes and how attitudes affect their intention towards formal recycling.

**Methods:**

A questionnaire survey was administered to 427 permanent residents of Hefei, a pilot city for electric vehicle and battery recycling initiatives. Data were analyzed using Partial Least Squares Structural Equation Modeling to test the hypothesized pathways linking place attachment dimensions to sustainable attitudes and recycling intentions.

**Results:**

The findings reveal that nature bonding is the strongest predictor across all three attitudes, economic attitude exerts the most powerful direct effect on recycling intention, and the combined sustainable attitudes explain 44.9 % of the formal recycling intention.

**Discussion:**

These results demonstrate that stronger emotional and cognitive ties to one’s locality significantly enhance pro‑recycling attitudes, whereas attitudes affect the willingness to participate in formal power battery recycling channels. Effective power battery recycling campaigns in collectivist contexts should therefore move beyond generic appeals and leverage residents’ specific attachments to their community’s nature, economy, and social fabric. This study contributes to environmental psychology by integrating place‑attachment constructs into a reasoning‑based model of sustainable behavior, and offers actionable insights for municipal authorities, recycling firms, and community groups seeking to improve formal recycling rates and advance circular‑economy objectives in rapidly urbanizing regions.

## 1 Introduction

**Figure 1:**
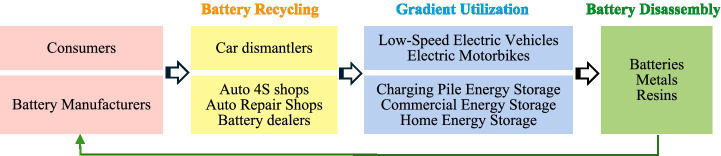
PBR process.

The power battery recycling (PBR) industry emerged globally following the European Union’s 2006 Directive on Waste Batteries and Accumulators. This industry specializes in processing used electric batteries from electric vehicles and other devices once their capacity falls below 70–80% (see Figure 1; Li J, et al., 2023). The PBR industry contributes to economic stability by recovering valuable metals and reducing reliance on mining (Zahoor et al., 2023), generates social benefits through workforce development programs and job creation (Liu and Yuan, 2023), and protects the environment through waste management (Zhao et al., 2024).

China’s systematic development of power battery recycling has progressed through four distinct policy-driven phases, a trajectory supported by scholarly analysis. The initial exploration (2012–2015) was catalyzed by the “Energy-saving and new energy vehicle industry development plan,” which first identified the strategic importance of battery recycling (Yang et al., 2021). This was followed by a phase of regulation formulation (2015–2018). The “Technical policy for the recycling and utilization of electric vehicle power batteries” was used to guide companies in the rational design, production, and recycling of electric vehicle power storage batteries, thereby establishing a preliminary regulatory scaffold (Song, 2023). The subsequent policy promotion (2018–2021) phase intensified efforts with pilot programs in 46 cities, including Hefei, to formalize industry channels. “Notice on piloting the recycling and utilization of new energy vehicle power batteries” was issued by the Ministry of Industry and Information Technology (MITT; Yao et al., 2022). Finally, the rapid market expansion (2021-present) phase is being driven by the impending wave of retired batteries, leading to the “New Energy Vehicle Industrial Development Plan 2021–2035” and market mechanisms that have accelerated the industry’s scale and sophistication (Update P, 2021).

**Figure 2:**
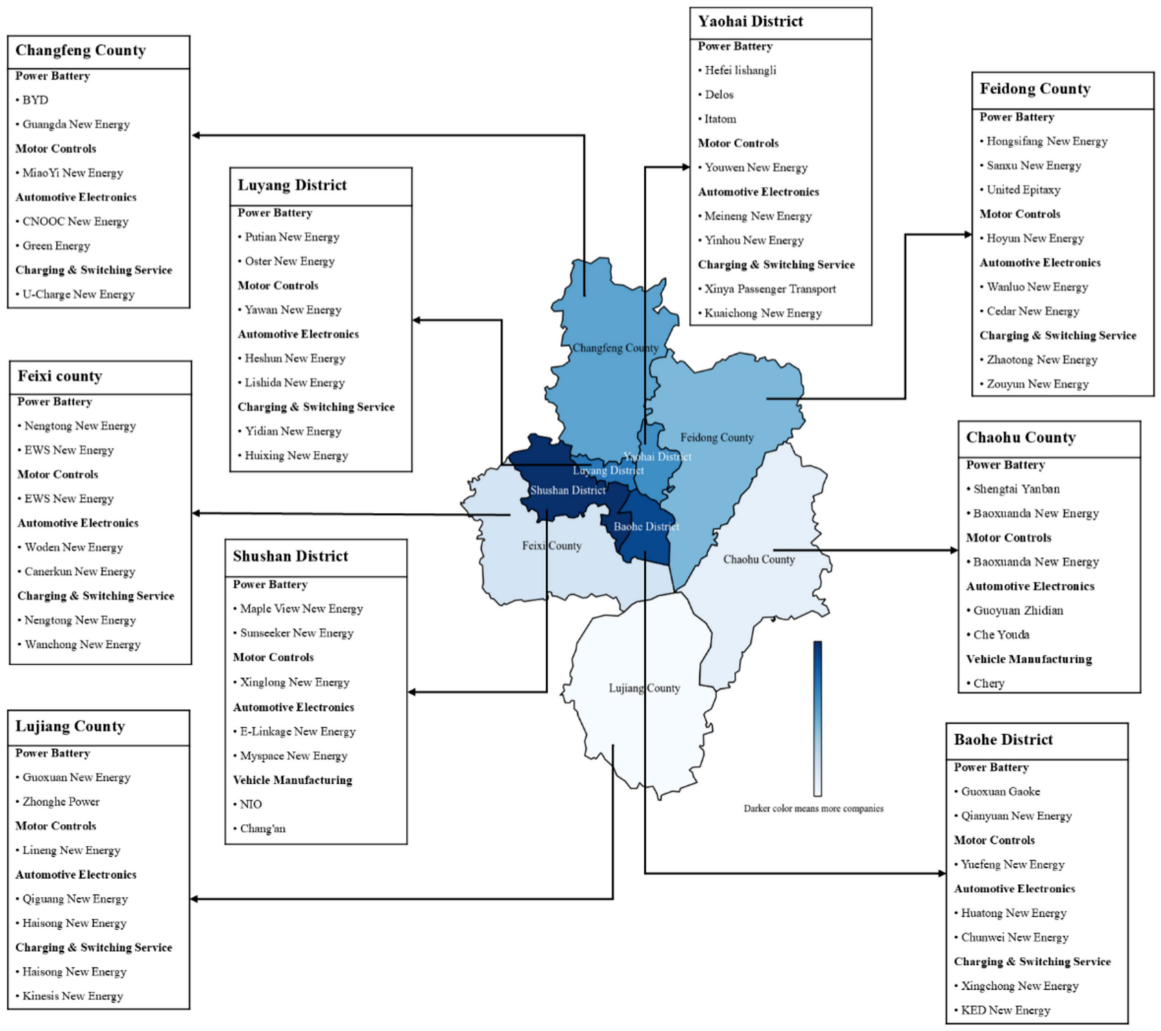
Companies in the electric vehicle industry chain in Hefei.

Hefei, a pilot city for PBR and new energy vehicles (NEVs), exemplifies this strategy. In 2020, the city’s investment of RMB 9 billion (45% of municipal funds) in NIO (a leading electric vehicle manufacturer) and similar bold investments catalyzed its NEV industry, which later generated RMB 300 billion in annual sales and accounted for 25% of China’s automobile exports (Zuo, 2022). This success fostered a trillion-yuan automotive cluster encompassing battery manufacturers (see Figure 2), driving a 10.2% increase in disposable income per capita (China’s highest) while accelerating NEV adoption and subsequent battery retirement. However, challenges still persist. Despite China’s 2017 pilot program in 46 cities, including Hefei, formal recycling rates remained below 25% by 2023 (Peng, 2024). The impending retirement of early-stage battery products, projected to reach 380.5 GWh (RMB 150 billion) by 2030, exacerbates existing systemic issues (Lee, 2023). Market observations suggest that a prevailing seller’s market favors informal recyclers, particularly small workshops that benefit from lower environmental compliance costs, thereby marginalizing formal recycling channels (Wang, 2023). These market dynamics highlight the pressing need to understand the determinants of formal recycling intentions, which is a crucial research and policy priority for realizing circular economy principles and achieving threefold sustainability goals.

Although formal recycling intentions have gained attention, current PBR research remains sectorally fragmented (see Table 1). For example, government-focused studies emphasize policy frameworks and regulatory systems (Lin et al., 2023), corporate-oriented research prioritizes recycling efficiency and profitability (Zheng et al., 2022), and academic work often highlights human capital and technological advancement. This institutional focus has largely overlooked a critical behavioral agent, the residents of collectivist societies. According to Hofstede’s cultural dimensions theory, collectivist cultures emphasize group harmony, community relationships, and shared responsibilities, which fundamentally shape psychological processes and behavioral motivations (Brewer and Venaik, 2011). These cultural characteristics suggest that place attachment mechanisms may operate differently than in individualistic Western contexts, where most existing research is conducted. Residents constitute the initial link in the recycling chain; however, their cognitive processes in collectivist settings remain understudied due to methodological complexities in capturing the multidimensional nature of psychological constructs like place attachment and its relationship to recycling behaviors (Liu et al., 2023), creating a clear need for deeper investigation. These challenges primarily stem from the need for sophisticated structural equation modeling to analyze complex relationships and the difficulty in developing culturally appropriate measurement instruments for collectivist contexts, creating a clear need for deeper investigation. The opportunities and challenges of harnessing place attachment (PA) to foster PBR remained uncovered. Furthermore, a recent study has acknowledged the psychological dimension of recycling, demonstrating that PA mediates the relationship between policy and low-carbon behaviors (Lin et al., 2024; Lin et al., 2025). However, these studies treat PA as a single monolithic construct. In the broader PBR literature, PA is either collapsed into a unitary variable (Wan et al., 2021) or only isolated dimensions are examined (Nketiah et al., 2022). The literature has failed to capture how the distinct facets of PA collectively and differentially operate through specific cognitive pathways to influence intention. Consequently, there is a lack of nuanced, multidimensional conceptualization of PA within the PBR context and an absence of empirical evidence on how each PA component influences the three attitudinal antecedents of recycling. Most prior work is situated in Western or policy-centric settings, while the motivational role of PA among residents in collectivist cultures such as China remains largely unexplored.

**Table 1:** Selected studies on recycling intention.

Reference	Type of research	Place of research	Industry	Theory	Findings
Yin et al. (2014)	Quantitative survey	China’s mainland	Mobile phone recycling	Extended producer responsibility	Region, education level, and monthly income influence consumers’ recycling intention and willingness to pay, so local governments and educational institutions bear responsibility for recycling initiatives.
Echegaray and Hansstein (2017)	Case study	Brazil	Electronic waste recycling	Theory of planned behavior	The intention-behavior gap in recycling behavior, particularly skewed among higher-income groups, underscores the need for government enforcement of regulations.
Moh (2017)	Comparative Study	Finland, Turku	Solid waste recycling	Theory of planned behavior	Stringent enforcement and regulatory actions promote recycling intention.
Shevchenko et al. (2019)	Literature review	Nil	Electronic waste recycling	Extended producer responsibility	Although the electronic bonus card system improves recycling intention, new economic incentives can be used to increase consumer collection rates.
Zheng et al. (2022)	Simulation analysis	Nil	Electronic waste recycling	Evolutionary game theory	It is beneficial to build its own recycling channel and adopt a differentiated price strategy, but consumers’ preference for the e-platform negatively affects recycling quantities.
Tang et al. (2023)	Quantitative survey	China	Electric vehicle battery recycling	Nil	Perceived behavior control, economic incentive, subjective norm, and self-identity influence the recycling intention.
Lin et al. (2025)	Quantitative survey	China	Nil	Theory of planned behavior	Place attachment and low-carbon behavioral intention indirectly mediate the relationship between information policy and low-carbon behaviors.

Addressing these gaps is essential for developing behavior-centered theories that can guide targeted interventions and policy measures. By dissecting PA into its constituent dimensions and testing their direct effects on distinct recycling attitudes, this study provides a granular psychological explanation of how residents’ attachment to place motivates formal recycling participation. The findings will enrich environmental psychology theory, offer transferable insights for cities worldwide seeking to strengthen circular economy pathways, and generate evidence-based recommendations aligned with SDG 11 (sustainable cities) and SDG 12 (responsible consumption). In summary, this study addresses the research gap by examining a specific research question in greater detail: How does PA serve as a motivational factor (‘reasons for’) influencing residents’ intention to participate in formal recycling? To address the research question, this research has four primary objectives.

1. Develop a comprehensive theoretical model and empirically validate the model through quantitative analysis.
2. Examine the relationship between three place attachment dimensions (place identity, social bonding, and natural bonding) and the (economic, social, and environmental) attitudes of Hefei residents.
3. Investigate how each attitudinal dimension predicts residents’ intention to engage in formal recycling.
4. Derive evidence-based recommendations for policymakers and practitioners to enhance formal recycling participation.

By addressing these goals, this study advances environmental psychology, clarifies the multifaceted role of PA in sustainable urban behaviors, and offers actionable strategies for fostering PBR in Hefei and comparable urban contexts. The subsequent sections present the literature review, data analysis, and concluding recommendations.

## 2 Review of literature

### 2.1 Theoretical foundations

**Figure 3:**
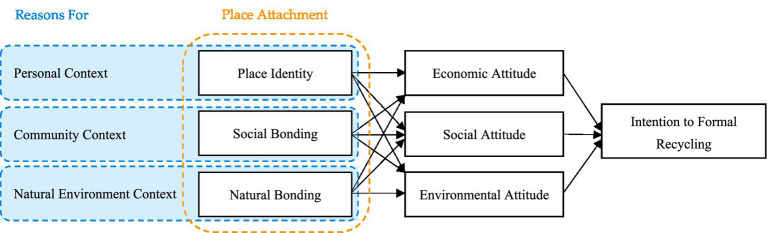
Framework.

The proposed framework (Figure 3) examines seven constructs through the lens of behavioral reasoning theory (BRT). This model uniquely positions PA as an antecedent to attitudes that shape recycling intentions, addressing critical sustainability demands. While existing research has investigated sustainable urban behavior, the application of BRT to PA-recycling intention relationships remains unexplored (Mitrofanova et al., 2021), which is a significant theoretical gap that this study addresses. This approach advances both methodological consistency and theoretical refinement in understanding recycling decisions.

China, in particular, is often regarded as a context-rich environment because of its cultural diversity, historical depth, economic variability, social structures, and political landscape. These factors create a dynamic and multifaceted context in which understanding the nuances of the environment is crucial for effective communication and decision-making (Jiang and Gossage, 2025). While the theory of planned behavior (TPB) has been widely applied in the study of consumer behavior, BRT presents a more suitable theoretical lens for investigating complex real-world issues, such as recycling in context-rich environments (Ahmed et al., 2025). Unlike TPB’s focus on attitudes, norms, and perceived behavioral control, the BRT integrates interdisciplinary insights from psychology, sociology, and behavioral economics, providing a deeper analysis of the cognitive and social factors shaping recycling intentions.

Additionally, the BRT framework considers various drivers of behavior, including individual reasons, attitudes, and intentions, offering a more holistic view of the factors that influence recycling (Mohanty et al., 2025). In this context, place attachment is conceptualized as a “reason for” variable that shapes individuals’ recycling perceptions through emotional and psychological ties to the environment. Recent research has cemented the importance of place attachment as a key psychological mechanism driving low-carbon behaviors in residential contexts. Notably, studies in China have demonstrated that place attachment serves as a critical mediator, translating policy satisfaction into pro-environmental action (Lin et al., 2024) and channeling the impact of energy policies on residential low-carbon behaviors (Lin et al., 2025). These findings confirm that residents’ emotional and social bonds with their community are pivotal in motivating sustainable practices. This study builds upon this foundation by deconstructing the broad concept of place attachment into its core dimensions, such as place identity, social bonding, and nature bonding, to investigate their specific, disaggregated influence on the attitudes that ultimately shape intention. This provides a more nuanced understanding of how different facets of attachment operate within the reasoning process that leads to formal recycling behaviors. In other words, place attachment influences how individuals emotionally connect with their environment, which can shape their commitment to sustainable practices, such as recycling. Furthermore, BRT highlights how economic, social, and environmental attitudes directly influence recycling intentions, as individuals rationally weigh the costs, benefits, and societal impacts.

Therefore, the application of BRT in this study provides critical insights into the complexities of recycling decision-making. The framework yields robust and actionable findings that advance both behavioral understanding and environmental protection efforts. By employing BRT strengthens both the theoretical foundation for sustainable behavior research and practical strategies to promote recycling.

### 2.2 Hypothesis development

Battery manufacturers are increasingly focusing on promoting recycling in accordance with sustainability goals. Place identity, social bonding, and nature bonding are integral aspects of place attachment that shed light on how individuals connect with their surroundings. These dimensions encompass various aspects of attachment, including physical places, social communities and natural landscapes. Although place attachment influences decision-making, attitudes reflect individuals’ perspectives on the economy, society, and the environment.

#### 2.2.1 Place identity and sustainable attitudes

Place identity (PI) refers to the emotional and psychological connection individuals have with a specific location, driven by a sense of belonging, attachment, and identification with that location. This connection often stems from personal experiences, memories, and cultural significance associated with a place (Nketiah et al., 2022). Place identity significantly influences individuals’ attitudes toward economic activities within their community. This emotional investment fosters feelings of ownership over local resources and pride in economic achievements, thereby influencing support for growth initiatives. In addition, place attachment promotes trust and cooperation, facilitating collaborative efforts toward economic advancement. Collectively, a stronger sense of PI correlated with more positive economic attitudes, suggesting its importance in promoting a community’s prosperity.

People with strong PI feel deeply connected to their community and promote solidarity and support among its inhabitants. This encourages active community participation, promotes cooperation to overcome challenges and improve well-being, strengthens social bonds, and fosters a positive attitude (Nketiah et al., 2022). Place attachment facilitates the formation of social support networks, promoting mutual assistance and companionship among residents, leading to a sense of belonging and well-being, and ultimately to positive social attitudes and community cohesion (Fornara et al., 2020). Furthermore, shared experiences, values, and cultural norms within the community uphold mutual respect, promote social harmony, and encourage positive attitudes.

PI signifies a profound emotional attachment to the natural features of a location, such as parks and rivers, and encourages people to cherish and protect their environment. This bond fosters a positive environmental attitude centered on conservation and sustainability, as individuals feel a personal responsibility and stewardship to preserve their local surroundings, motivating proactive behaviors, such as recycling (Faccioli et al., 2020). Additionally, individuals deeply connected to their sense of place are more attuned to environmental changes, motivating them to enhance their surroundings for the good of their lives and their communities. This strong place attachment fosters community engagement in environmental efforts, promoting cooperation to achieve sustainability goals, such as creating green spaces and reducing waste.

*H*_1_: There is a positive relationship between place identity and sustainable attitudes.

*H*_1a_: There is a positive relationship between place identity and economic attitude.

*H*_1b_: There is a positive relationship between place identity and social attitude.

*H*_1c_: There is a positive relationship between place identity and environmental attitude.

#### 2.2.2 Social bonding and sustainable attitudes

Social bonding (SB) refers to the connections that individuals develop within a community associated with a specific location. It involves feelings of solidarity, mutual support, and camaraderie among individuals who share a common attachment to a place (Wan et al., 2021). Trust and cooperation improve perceptions of local economic entities and initiatives, whereas information sharing influences views on the regional economy (Leimar and Bshary, 2024). Additionally, SB fosters a shared identity, promotes support for economic progress, and reinforces social standards related to prosperity. It also reinforces social norms and values related to economic success, influencing people to adopt positive economic attitudes to promote social cohesion.

SB creates a sense of belonging and shared identity among community members, leading to shared values, norms, and beliefs, and promoting cooperation, empathy, and mutual support (Triantafyllidis and Darvin, 2021). It also facilitates interactions within the community, providing opportunities for individuals to learn from and empathize with others’ experiences and perspectives. These interactions promote understanding, tolerance, and acceptance of diversity, shaping individuals’ social attitudes toward inclusivity and social justice. Finally, SB builds trust and reciprocity among community members, encouraging collective action and collaboration to address social issues and challenges. In general, SB fosters positive social attitudes through community cohesion and solidarity.

SB within communities contributes to positive environmental attitudes through shared values, collective stewardship, social learning and supportive networks. When individuals feel connected to their community, they are more likely to adopt environmental values, engage in collective environmental actions, learn from others, and receive support for pro-environmental behavior (Triantafyllidis and Darvin, 2021). This cultivates a sense of responsibility for the environment and encourages sustainable practices in the community.

*H*_2_: There is a positive relationship between social bonding and sustainable attitudes.

*H*_2a_: There is a positive relationship between social bonding and economic attitude.

*H*_2b_: There is a positive relationship between social bonding and social attitude.

*H*_2c_: There is a positive relationship between social bonding and environmental attitude.

#### 2.2.3 Nature bonding and sustainable attitudes

Nature bonding (NB) refers to the emotional connection individuals feel with natural environments, encompassing awe, appreciation, and harmony with nature. Those with a strong connection to nature often exhibit environmental stewardship and support eco-friendly practices and industries. Furthermore, natural beauty attracts tourists and stimulates local economies through activities such as ecotourism and outdoor recreation. Communities anchored to nature can practice sustainable resource management, leading to economic benefits and improved livelihood. NB also fosters innovation in green technologies, creating job opportunities and contributing to positive economic attitudes (Hernández et al., 2020).

Nature-based group activities strengthen social bonds and foster friendships through shared experiences. These collective interactions build communities and camaraderie, enhancing social cohesion. Research shows that such nature engagement also improves mental well-being and promotes positive social attitudes (Nketiah et al., 2022). Moreover, nature provides opportunities for social gatherings and events, such as outdoor festivals or conservation projects, which can bring people together and foster a sense of belonging (Wan et al., 2021). Furthermore, involvement with nature can promote empathy and concern for others because experiencing the beauty and interconnectedness of the natural world can cultivate a sense of compassion and altruism.

NB promotes a positive environmental attitude by deepening appreciation of the natural world and promoting stewardship and responsibility for environmental resources (Nketiah et al., 2022). Through firsthand experiences and outdoor activities, people develop awareness of environmental issues and advocate for conservation efforts. NB also encourages sustainable behaviors and evokes feelings of awe and interconnectedness, motivating individuals to care for the planet and its ecosystems. Given their role in promoting awareness, empathy, and action for environmental conservation, the following hypothesis was proposed:

*H*_3_: There is a positive relationship between nature bonding and sustainable attitudes.

*H*_3a_: There is a positive relationship between nature bonding and economic attitude.

*H*_3b_: There is a positive relationship between nature bonding and social attitude.

*H*_3c_: There is a positive relationship between nature bonding and environmental attitude.

#### 2.2.4 Sustainable attitudes and intention to formal recycling

Economic attitude (ECA) refers to beliefs, perceptions, and feelings about financial matters, market dynamics, and government policies, covering wealth, income distribution, taxation, economic growth, and employment opportunities (Abadi, 2023). Drawing on the PB, individuals with positive ECA toward formal recycling view it as advantageous, resulting in cost savings, resource preservation, and economic benefits (Promalessy et al., 2025). They weigh the costs against the benefits of participating in recycling and support the policies that promote formal recycling. This cost–benefit calculus is also central to rational choice theory (Scott, 2000), which posits that individuals select the option that maximizes expected utility. Previous research suggests that economic considerations significantly influence environmental behaviors, particularly in recycling contexts where perceived economic benefits strongly predict participation intentions (Daoud et al., 2025). Viewing formal recycling as an investment in sustainable practices, they recognized its potential economic value in terms of resource conservation and revenue generation from recycled materials (Li C, et al., 2023). Consequently, individuals with a positive ECA are more likely to engage in formal recycling.

Social attitude (SA) refers to an individual’s beliefs, opinions, and feelings about social issues, norms, and behaviors within a community. This includes attitudes toward topics such as social justice, equality, diversity, interpersonal relationships, and community participation (Abadi, 2023). In TPB, social attitude captures perceived social pressure to perform a behavior, while normative conduct theory emphasizes that compliance with social attitude can be a primary driver of action, especially in collectivist cultures, where maintaining harmony and reputation is paramount (Cialdini et al., 1990). Grounded in TPB, SA influences the intention to formalize recycling programs through various mechanisms (Zuo et al., 2025). Individuals with favorable SA may view recycling as socially responsible and aligned with community values (Baeshen, 2025). Social identity theory suggests that when recycling is framed as a marker of “good citizen” status, individuals with strong identification with their community are more motivated to act in ways that reinforce that identity. They may feel compelled to recycle to conform to social norms or maintain their reputation (Abadi, 2023), particularly in collectivist cultural contexts, where social harmony and group conformity are highly valued. Additionally, positive SA fosters solidarity and encourages collective efforts toward environmental goals (Taouahria, 2024). It shapes perceptions of the social benefits linked to recycling, thereby enhancing community well-being and the quality of life. Based on this theoretical foundation, positive SA bolsters the intention to recycle.

Environmental attitude (ENA) refers to an individual’s sentiments and values regarding environmental preservation, sustainability, and resource management. This includes views on issues such as pollution, climate change, and ecological conservation (Abadi, 2023). The TPB positions attitude toward behavior as a direct antecedent of intention, and the new ecological paradigm operationalizes a worldview that emphasizes the interdependence of human activity and natural systems (Dunlap et al., 2000). Research in environmental psychology suggests that environmental attitudes serve as fundamental predictors of recycling behavior, with evidence confirming moderate to strong relationships across different populations (Promalessy et al., 2025). Building on TPB and the new ecological paradigm (Dunlap et al., 2000), positive ENA is strongly correlated with the intention to participate in formal recycling efforts across diverse cultural contexts. Individuals with such attitudes perceive recycling to be vital for conserving natural resources and mitigating environmental harm (Puzzo and Prati, 2024). They acknowledge recycling’s benefits in waste reduction and pollution prevention, feeling personally responsible for environmental stewardship and ready to fulfill their ethical obligations through pro-environmental behaviors (Islam et al., 2025). Consequently, positive ENA drives the intention of people to participate in formal recycling, aligning their behaviors with their environmental values and principles.

*H*_4_: There is a positive relationship between sustainable attitude and intention to formal recycling.

*H*_4a_: There is a positive relationship between economic attitude and intention to formal recycling.

*H*_4b_: There is a positive relationship between social attitude and intention to formal recycling.

*H*_4c_: There is a positive relationship between environmental attitude and intention to formal recycling.

## 3 Methodology

**Figure 4:**
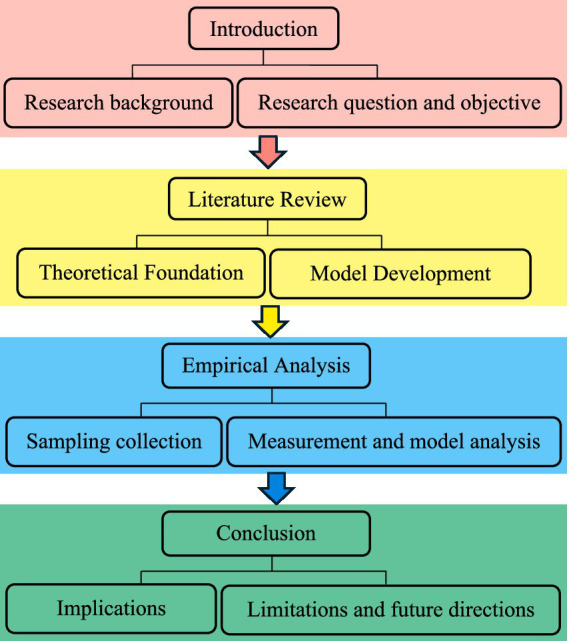
Research process.

The present research utilizes a positivist philosophy and a deductive approach with a cross-sectional quantitative design to test 12 hypotheses based on established theories. The research design was organized into various phases, as shown in Figure 4. First, research questions are derived from the background analysis. Second, a literature review synthesizes existing theories to develop a conceptual model. Third, a survey is conducted to test hypotheses; and fourth, the findings are discussed, highlighting implications and future research directions. In particular, attention was given to analyzing the reliability and validity of the measurement model to ensure that the observed variables accurately reflected the underlying constructs. Reflective model analysis further examines the relationships between constructs and indicators, validating the operationalization of the theoretical principles. This structured methodology systematically addressed the research objectives and strengthened the robustness of the findings.

### 3.1 Sampling collection

Hefei serves as a prime sample of China’s burgeoning new-energy vehicle (NEV) sector, which benefits its permanent residents through industrial expansion and urban development. Despite China’s overall population decline, Hefei’s permanent resident population reached 9.853 million in 2023, representing a 2.23% increase, the highest growth rate among Chinese cities with a GDP of RMB 26 trillion (Wang, 2024). The city’s sizable local market and the presence of major manufacturers such as NIO, BYD, Geely, Volkswagen, and Chery ensure that the latest NEVs are displayed continuously in shopping centers throughout the year. Visitors to these showcases are likely to possess a strong interest in electric vehicles and considerable knowledge of power-battery recycling, making them ideal respondents for this study.

Given the time constraints and the absence of an easily accessible sampling frame for Hefei’s permanent residents with formal recycling intentions, a non-probability sampling strategy was employed, targeting the showrooms where potential respondents could be reached more readily. Data were gathered from February 3 February to 25 February 2025, coinciding with the Chinese New Year celebrations. During this period, most migrant workers and university students returned to their hometowns for family reunions, a longstanding and customary practice that has endured for millennia within the traditional, collectivist family culture of China. This timing was strategically selected to access permanent residents with established connections to Hefei, which aligns with our research focus on place attachment. This timing was deliberately chosen to capture permanent residents who maintain strong ties to Hefei, aligning with the study’s focus on place attachment. Consequently, the final sample was predominantly composed of Hefei’s permanent residents.

The offline questionnaire administered at the vehicle showcases enabled researchers to interact directly with participants and clarify items in Mandarin or local dialects, which likely yielded higher response rates and richer feedback than an online survey would have produced. This independent study also underwent a comprehensive review to ensure compliance with ethical standards and to protect respondent anonymity, even though formal ethical approval from the university or the shopping-center management was not required. The sample size was determined according to the minimum guideline of 10 observations per indicator for partial least-squares structural equation modeling and confirmatory factor analysis (Hair et al., 2014), resulting in a required minimum of 270 respondents (27 indicators × 10). Of the 700 hard-copy questionnaires distributed, 427 valid responses were obtained from local permanent residents, yielding a 61% response rate that satisfies the statistical power requirements of our analytical approach. The final sample characteristics indicate a predominance of males (59%), aged 26–35 years (37%), married (62%), employed (72%), with bachelor’s degrees (34%), a monthly income of RMB 5,001–7,500 (68%), a family size of three (39%), and an electric car (53%). These demographics reflect both the methodological strategy and the typical profile of electric-vehicle enthusiasts in Hefei.

### 3.2 Measurement

The first part of the questionnaire investigated the respondents’ demographic characteristics, and the second part comprised seven variables from the research framework. Adapting established measurement instruments ensures validity and consistency and reduces bias in research. Using rigorously tested scales allows for reliable measurements and facilitates comparisons across studies, saving time and enhancing the credibility of the outcomes. Specifically, a three-item scale was adapted to measure SA (Abadi, 2023), while six 4-item scales were adapted to measure PI (Plunkett et al., 2019), SB (Isa et al., 2020), NB (Strzelecka et al., 2017), ECA (Abadi, 2023), ENA (Abadi, 2023), and INT (Li C, et al., 2023), as shown in Appendix 1. The 27 items were measured using a five-point Likert scale, with options ranging from 1 (strongly disagree) to 5 (strongly agree). Furthermore, to improve content validity and make it more relevant to the target population, comments from three professors working at different universities were integrated into the final version of the questionnaire.

## 4 Data analysis

### 4.1 Measurement model assessment

Following the guidelines introduced by previous research (Hair et al., 2014), factor loading was used to assess reliability at the item level with a threshold of 0.7, while Cronbach’s alpha (CA) and composite reliability (CR) were used to assess reliability at the construct level with a threshold of 0.7. Cross-loadings complement factor loadings, CA, and CR by providing additional evidence that the indicators uniquely and reliably measure their intended constructs. Cross-loadings ensure that each indicator loads more strongly on its intended construct than on any other construct in the model. This is critical for establishing indicator reliability and discriminant validity at the item-level. Furthermore, to quantify the amount of variance captured by the items of a construct relative to the amount of variance due to measurement error, average variance extracted (AVE) was used to evaluate convergent validity with a threshold of 0.5. As indicated in Appendix 2, the thresholds for factor loading, CA, CR, and AVE were met in this investigation. Furthermore, to examine whether the constructs in a model are distinct from each other, the Fornell-Larker criterion was utilized to assess the discriminant validity. As indicated in Appendix 3, the items exhibited discriminant validity, with primary loadings consistently surpassing cross-loadings. As indicated in Appendix 4, the square root of the AVE of each construct is greater than the correlations between that construct and all other constructs in the model. Additionally, the Heterotrait-Monotrait (HTMT) ratio of correlations provides a more stringent test for discriminant validity by examining whether the relationships between constructs are stronger within constructs than between constructs. A threshold of 0.85 was met, as shown in Appendix 5.

The overall fit of the partial least squares structural equation modeling (PLS-SEM) model was assessed using approximate fit indices. The results, presented in Table 2, indicate an acceptable model fit. First, the Standardized root mean square residual (SRMR) is a key metric for evaluating the average discrepancy between the observed correlations and the model-implied correlations. Both SRMR values (0.062, 0.077) are below the recommended threshold of 0.08, indicating a good fit and suggesting that the model does not contain substantial path model misspecification (Asparouhov and Muthén, 2018). The normed fit index (NFI), which compares the chi-square value of the proposed model to that of a null model, was above the ideal benchmark of 0.90 (Hair et al., 2014), indicating a reasonable level of fit. The significant chi-square statistic (χ^2^ = 1465.140, *p* < 0.001) is common in complex models with large sample sizes. This result does not, by itself, invalidate the model. The squared Euclidean distance (d_ULS; 1.435 → 2.263) and the geodesic distance (d_G; 0.536 → 0.618) were within the range typically regarded as satisfactory for PLS-SEM, as a previous study treated values around 0.5–0.6 as indicative of a modest distance between the empirical covariance matrix and the model-implied matrix (Pawaskar and Nattuvathuckal, 2024).

**Table 2:** CB-SEM or PLS-SEM selection.

Criterion	CB-SEM	SEM-PLS
Research aim	Parameter-oriented	Prediction-oriented
Approach	Covariance-based	Variance-based
Sample size	Large	Small
Relation to theory	Confirmatory	Predictive
Software	AMOS, LISREL, MPlus	Smart-PLS, Warp-PLS, PLS-Graph

### 4.2 Structural model analysis

Path coefficients, coefficient of determination (*R^2^*), effect size (*f^2^*), and predictive relevance (*Q*^2^) were used for structural model analysis. These values were obtained by PLS-SEM using the SmartPLS software. Compared to covariance-based structural equation modeling (CB-SEM), PLS-SEM is preferred for predictive modeling, smaller samples, nonnormal data, and complex models, making it a versatile tool in social sciences and business research (Appendix 6).

**Figure 5:**
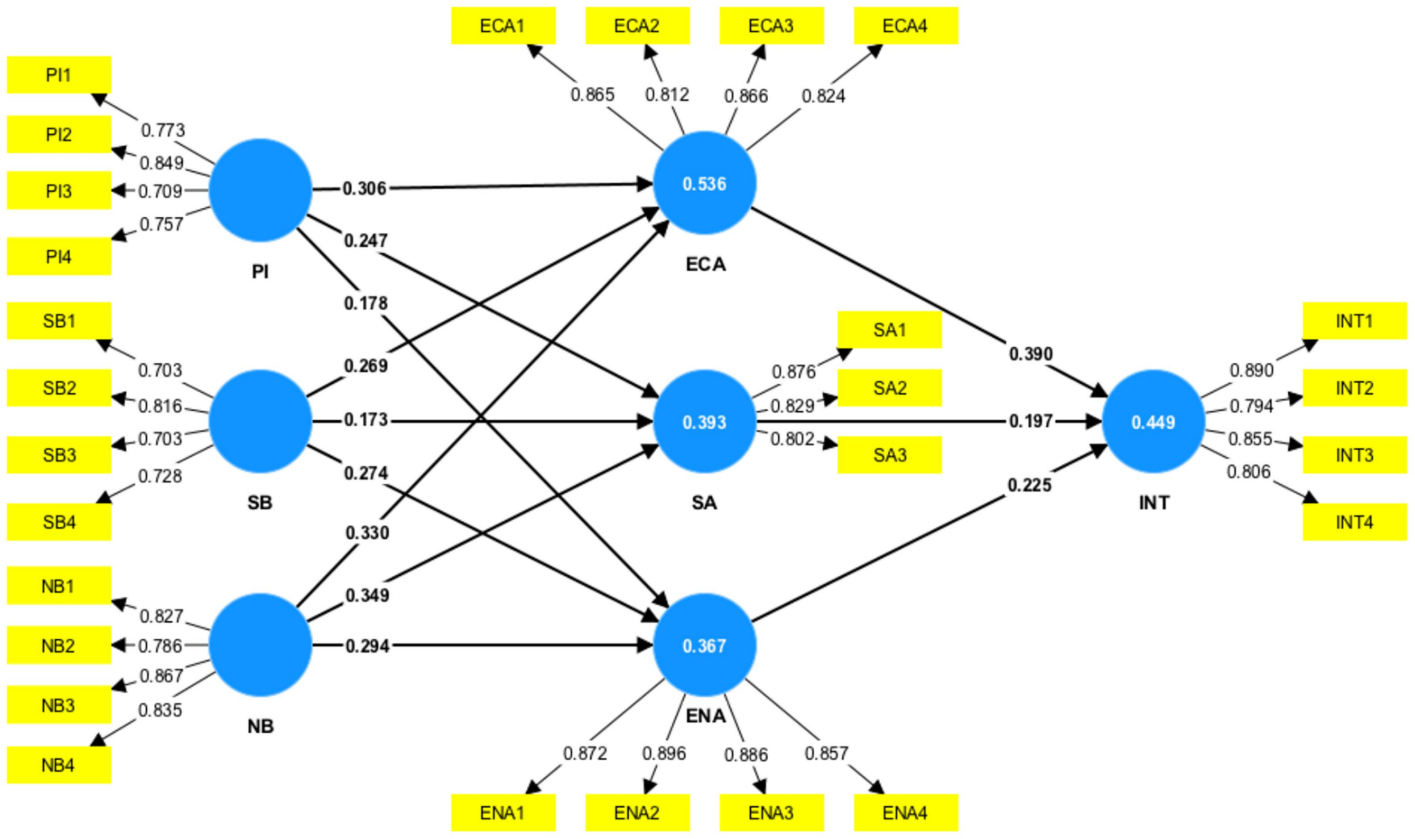
Measurement model with item loading, path coefficient, and *R^2^* values.

Figure 5 and Table 3 show that PI (*β* = 0.306, *p* < 0.05) positively affects ECA, supporting H_1a_. PI (*β* = 0.247, *p* < 0.05) positively affects SA, supporting H_1b_. PI (*β* = 0.178, *p* < 0.05) positively affects ENA, supporting H_1c_. SB (*β* = 0.269, *p* < 0.05) positively affects EA, supporting H_2a_. SB (*β* = 0.173, *p* < 0.05) positively affects SA, supporting H_2b_. SB (*β* = 0.274, *p* < 0.05) positively affects ENA, supporting H_2c_. NB (*β* = 0.173, *p* < 0.05) positively affects ECA, supporting H_3a_. NB (*β =* 0.173, *p* < 0.05) positively affects SA, supporting H_3b_. NB (*β* = 0.173, *p* < 0.05) positively affects ENA, supporting H_3c_. EA (*β* = 0.173, *p* < 0.05) positively affects INT, supporting H_4a_. SA (*β* = 0.173, *p* < 0.05) positively affects INT, supporting H_4b._ EA (*β* = 0.173, *p* < 0.05) positively affects INT, supporting H_4c_.

**Table 3:** Direct effects.

Relationship	*β*	Standard deviation	T statistics	*R^2^*	*f^2^*	*Q* ^2^	*p*-values	Decision
PI ➔ ECA	0.306	0.040	7.683	0.536	0.136	0.373	0.000	Support
SB ➔ ECA	0.269	0.044	6.187		0.106		0.000	Support
NB ➔ ECA	0.330	0.036	9.045		0.166		0.000	Support
PI ➔ SA	0.247	0.048	5.160	0.393	0.068	0.268	0.000	Support
SB ➔ SA	0.173	0.052	3.338		0.034		0.001	Support
NB ➔ SA	0.349	0.043	8.105		0.142		0.000	Support
PI ➔ ENA	0.178	0.047	3.804	0.367	0.034	0.278	0.000	Support
SB ➔ ENA	0.274	0.049	5.601		0.081		0.000	Support
NB ➔ ENA	0.294	0.044	6.638		0.097		0.000	Support
ECA ➔ INT	0.390	0.047	8.380	0.449	0.171	0.307	0.000	Support
SA ➔ INT	0.197	0.042	4.663		0.053		0.000	Support
ENA ➔ INT	0.225	0.044	5.157		0.061		0.000	Support

*R^2^* represents the goodness of fit of the regression model. The combined influence of PI, SB, and NB accounts for 53.6% of the variance in ECA, 39.3% of the variance in SA, and 36.7% of the variance in ENA, respectively. Similarly, 44.9% of the variance in INT is explained by the combined influence of ECA, SA, and ENA. As a result of these *R^2^* values, the research model demonstrated a substantial capacity to explain the variance in ECA and a moderate capacity to explain the variance in SA, ENA, and INT.

*f^2^* measures the magnitude of the effect of independent variables on the dependent variable (Hair et al., 2014). NB (*f^2^* = 0.166) has a medium effect in explaining the variance in ECA, while ECA (*f^2^* = 0.171) has a medium effect in explaining the variance in INT. In comparison, the effect sizes of the rest of the independent variables on the dependent variables are weaker.

*Q*^2^ is closely related to *R^2^* but specifically focuses on the predictive capability of the model for new data. Assessed using Stone-Gasser’s Q-Square approach, the consistently positive Q^2^ values across all endogenous constructs (range: 0.268–0.373) provide empirical evidence for the model’s predictive validity in the context of power battery recycling. These findings suggest the specified structural relationships effectively capture the underlying behavioral mechanisms.

## 5 Discussion

Responding to the call of a previous study (Wan et al., 2021), this research established that PA serves as one of the ‘reasons for’ influencing residents’ INT within Hefei’s growing PBR ecosystem. Hefei’s strategic investments in NEV and PBR infrastructure also provided a solid foundation for studying this dynamic. In response to research objective one, the successful validation of a PLS-SEM model confirms the utility of a multidimensional place attachment framework within BRT.

### 5.1 The direct role of place attachment in shaping attitudes

Testing of H_1_ to H_3_ achieved research objective two, demonstrating significant positive relationships between PA and attitudes. These findings challenge conventional economic rationality models by showing that emotional connections to place can fundamentally reshape economic evaluations of sustainability initiatives. In particular, the results of H_1_ validated the positive impact of PI on ECA, SA, and ENA, consistent with a previous study (Nketiah et al., 2022), underscoring the importance of a strong emotional and cognitive connection to one’s locality. This finding extends the existing literature by revealing the psychological mechanism through which place identity operates. When individuals derive their self-concept from their locality, they internalize sustainability issues as personal concerns. The particularly strong relationship between place identity and economic attitude provides novel theoretical insight, suggesting that in rapidly industrializing cities like Hefei, residents perceive green industry prosperity as intrinsically tied to their own well-being, thereby transforming abstract economic development into personally relevant outcomes. In Hefei, where municipal efforts have prioritized creating a cohesive industrial and social ecosystem around the NEV industry, place identity is reinforced by visible advancements in infrastructure, employment opportunities, and environmental improvements. It is therefore worthwhile to enhance place identity among residents through various approaches.

The results of H_2_ confirmed the role of SB in shaping residents’ ECA, SA, and ENA, consistent with previous studies. This extends the understanding of social influence beyond explicit norms to include community solidarity. It also challenges individual-centric models of attitude formation by demonstrating that community networks serve as conduits for transmitting and reinforcing sustainable values. The significant relationship between social bonding and environmental attitude indicates that in Chinese collectivist culture, shared responsibility for communal environments, fostered through trust and cooperation, motivates residents beyond individualistic environmental concerns. This suggests that environmental stewardship arises as much from social solidarity as from individual environmental concern, offering a culturally nuanced understanding of pro-environmental attitude formation that contextualizes Western individual-oriented models within collectivist cultural frameworks. Indeed, Hefei’s strong community networks and government-led programs promoting recycling awareness have likely contributed to the promotion of trust, collaboration, and shared responsibility among residents. SB is further enhanced by facilitating EV owner meet-ups or carpool programs to encourage interaction and camaraderie among residents interested in low-carbon transportation (Liu et al., 2024).

The validation of H3 establishes nature bonding as the most consistent predictor across all attitudinal dimensions. This finding significantly advances environmental psychology literature by demonstrating that emotional connection to nature generates not only environmental concern but also enhances perceptions of economic and social benefits. For example, residents who value Hefei’s natural environments better recognize the economic value of resource conservation, appreciate the social benefits of a clean environment, and feel a personal duty to mitigate environmental harm. The significant relationship with economic attitude shows that residents’ emotional bonds with natural environments enable them to recognize the tangible economic value of resource conservation, transforming abstract sustainability concepts into practical economic considerations. Furthermore, the particularly strong effect on social attitude extends previous research (Bonaiuto et al., 2002) by revealing a psychological mechanism through which nature appreciation fosters community-oriented values and shared environmental responsibility, thereby creating a virtuous cycle between environmental connectedness and social cohesion. These results collectively demonstrate that nature bonding cultivates comprehensive stewardship ethics where environmental, social, and economic considerations become mutually reinforcing priorities in sustainable decision-making.

### 5.2 The direct role of sustainable attitudes in shaping intention

Research objective three was achieved by validating the attitude-intention correlation in hypothesis H4, consistent with previous research (Abadi, 2023). The results for H4a identified economic attitude as the strongest predictor of recycling intention, providing a fundamental theoretical insight that challenges conventional environmental behavior models. This finding indicates that in emerging economy contexts such as Hefei, recycling intention reflects a pragmatic sustainability choice in which economic rationality outweighs environmental concerns. Moreover, this suggests that in such contexts, recycling intention is a pragmatic sustainability choice rather than purely an environmental behavior. The predominance of economic considerations in intention formation extends BRT by demonstrating the contextual dependency of “reasons,” revealing how developmental priorities can reshape the hierarchy of behavioral determinants. This theoretical advancement helps explain why residents’ practical concerns, such as monetary incentives, cost savings, and broader economic benefits, outweigh purely environmental motivations in their recycling decisions, thereby reframing our understanding of pro-environmental behavior in rapidly developing urban contexts.

The results of H_4b_ corroborated the positive impacts of SA on INT (Abadi, 2023), aligning with but substantially refining existing knowledge about normative influences in environmental behavior. This finding contributes to the social psychology literature by demonstrating that in collectivist societies as China, social attitudes function not merely as external pressures but as internalized values that directly shape behavioral intentions. The significant relationship suggests that social influence operates through cognitive evaluation processes rather than solely through normative compliance, revealing how community-driven initiatives and educational campaigns in Hefei have successfully transformed recycling into a collectively internalized responsibility rather than merely an externally imposed obligation. This theoretical advancement helps explain why social attitudes remain a significant predictor of intention even when economic considerations dominate, highlighting the complex interplay between different motivational factors in the formation of sustainable behavior.

The validation of H_4c_ establishes ENA as a significant, though secondary, driver of INT, consistent with previous studies (Li J, et al., 2023). This finding offers an important theoretical qualification to the environmental psychology literature by revealing that, while environmental concern is important, its influence may be contextually affected by other considerations in emerging economy settings. The results demonstrate a hierarchical ordering of attitudinal influences on sustainability behaviors, with economic pragmatism prevailing in contexts where immediate economic development priorities are prominent. This theoretical insight substantially refines our understanding of pro-environmental behavior by suggesting that environmental attitudes function within a complex motivational framework in which economic and social considerations may take precedence. Nevertheless, the significant coefficient confirms that ecological stewardship and increasing public awareness of environmental hazards remain crucial components in forming recycling intentions, highlighting the multifaceted nature of sustainable decision-making in urban environmental contexts.

Collectively, these findings from H_4_ advance theoretical understanding by revealing the complex interplay between different attitudinal dimensions in the formation of behavioral intention. The substantial explanatory power of the model demonstrates that considering the multidimensional nature of attitudes provides a more comprehensive framework for understanding sustainability behaviors than the single attitude approaches predominant in existing literature. This integrated perspective reveals how economic pragmatism, social responsibility, and environmental concern collectively, though not equally, contribute to behavioral intentions, with economic considerations emerging as the primary driver in this specific developmental context. The findings thus challenge reductionist models of environmental behavior while offering a more nuanced theoretical framework that accounts for the contextual hierarchy of motivational factors in sustainable decision-making.

Furthermore, this study identified contextual challenges that affect the application of psychological models in Hefei’s unique environment. The significant gap between residents’ demonstrated recycling intentions and the city’s actual formal recycling rate, which remains below 25 percent, reveals how structural barriers can substantially moderate the intention-behavior relationship. The prevalence of informal recycling workshops, combined with limited access to formal recycling channels, creates a market environment that undermines the effectiveness of psychologically based interventions. Additionally, the simultaneous retirement of large volumes of early-stage power batteries presents complex logistical and regulatory challenges, while the prevailing seller’s market model continues to incentivize environmentally unregulated practices. These findings extend BRT by demonstrating that even strongly held psychological motivations for action may be neutralized by countervailing structural and market forces, highlighting the necessity of addressing both psychological and contextual factors in promoting sustainable behaviors.

### 5.3 The advancement of behavioral reasoning theory

This research advanced BRT in several ways. First, by incorporating personal, community, and natural environment contexts, the framework acknowledges that individual behaviors, such as recycling, are influenced not only by personal beliefs but also by broader social and environmental factors. This expansion extends the theory to consider external influences, broadening its scope. Second, the inclusion of PI, SB, and NB provides greater theoretical nuance by illustrating how emotional connections to places and communities shape behavioral intentions through distinct psychological pathways. This offers more nuanced insights into how PA impacts environmental behaviors. Third, the study enhances the theory’s explanatory power by establishing direct relationships between three-dimensional attitudes and behavioral intentions, providing a more comprehensive understanding of how various attitudes interact in sustainable decision-making. Fourth, it strengthens the theory’s applicability to collectivist contexts by demonstrating how social attitudes and community relationships influence recycling behaviors through internalized values rather than merely external pressures. Furthermore, by bridging theory with concepts from environmental psychology, the framework deepens our understanding of how individual and collective environmental behaviors are formed and sustained. Finally, by identifying the reasons behind behaviors and the role of PA, policymakers can develop targeted interventions that align with the specific contexts of individuals, thus increasing the practical relevance of the theory. Collectively, these contributions provide a more comprehensive theoretical framework that accommodates a broader range of influences on recycling behaviors while enhancing the theory’s practical applicability in environmental behavior research across different cultural and economic contexts.

## 6 Conclusion, implications, and limitations

### 6.1 Conclusion

This research sheds light on the central role of PA in shaping residents’ INT in Hefei. By emphasizing the interplay between PI, SB, and NB as well as ECA, SA, and ENA, it provides a comprehensive framework for understanding recycling behaviors in collectivist societies. The findings underscore the need for targeted interventions that leverage residents’ emotional and cognitive connections to their locality while addressing structural barriers to formal recycling. Policymakers and stakeholders can use these insights to develop targeted interventions and policies for sustainable waste battery management. Initiatives such as community engagement events, educational programs, and recognition schemes can foster pride and encourage resident participation in formal recycling. By aligning managerial strategies and policy interventions with these findings, stakeholders can advance sustainable development goals and support the growth of the PBR industry in the area. Ultimately, this research contributes to understanding the sociopsychological factors that influence recycling intention, paving the way for more effective waste management strategies in cities such as Hefei and beyond. As Hefei continues to lead China’s new energy initiatives, the lessons learned from this study can inform broader efforts to promote sustainable waste management practices in emerging cities around the world.

### 6.2 Theoretical and managerial implications

This research advances BRT by demonstrating its applicability to PBR decision-making in urban contexts. By decomposing PA into PI, SB, and NB as distinct constructs and integrating three attitudinal dimensions, this research enhances BRT’s ability to explain complex decision-making processes. The empirical validation of these relationships in Hefei’s collectivist context addresses a critical gap in environmental psychology literature and challenges conventional one-dimensional approaches to PA. These findings highlight the necessity of interdisciplinary approaches that incorporate psychological, social, and spatial factors to better understand sustainability-related decision-making.

Building on China’s existing policy framework, the research findings suggest several specific enhancements to strengthen PBR systems. First, the extended producer responsibility (EPR) framework under the “Action plan for strengthening new energy vehicle power battery recycling system 2025” (Mengyang, 2025) can be improved by incorporating manufacturer-supported community engagement programs that strengthen PI. Rather than treating residents merely as policy targets, manufacturers could use EPR funds to support industry NEV. Such initiatives would strengthen PI by connecting residents emotionally to local industrial achievements, thereby enhancing their economic attitude toward recycling.

Second, the “Battery ID system 2024–2025” (Ge, 2025) presents an opportunity to enhance SB by integrating community engagement features. The tracking system could be expanded beyond technical monitoring to include neighborhood-level performance metrics, becoming a tool for building social networks and strengthening collective environmental responsibility. This approach would leverage SB mechanisms by creating visible social norms and fostering friendly competition between communities, addressing the SA dimension identified in this study. Thus, it would transform the technical tracking system into a tool for building social networks and strengthening collective environmental responsibility.

Third, the environmental standards in the “Comprehensive utilization standards of power battery 2024” (Zhongyang, 2025) should integrate NB principles by linking PBR with local environmental protection. Recycling enterprises could establish educational programs that demonstrate how proper battery disposal protects local ecosystems while organizing community cleanup events in partnership with local schools and environmental organizations. Recycling facilities could be required to partner with local communities on green space restoration initiatives funded by recycling proceeds. These practices would strengthen NB by creating tangible connections between recycling behaviors and local environmental benefits.

Furthermore, the findings support optimizing the placement of recycling infrastructure in locations with strong emotional significance to residents, such as community centers and public parks. This strategic placement leverages existing PA to increase recycling participation rates. Additionally, the study suggests enhancing NB through environmental education programs linked to existing recycling infrastructure. Recycling enterprises could partner with local schools and community organizations to establish educational programs that demonstrate how proper PBR protects local ecosystems. These programs should emphasize the connection between individual recycling behaviors and the preservation of Hefei’s natural environment.

### 6.3 Limitations and future directions

This study acknowledges several limitations that offer fruitful avenues for future research. First, the reliance on offline Hefei questionnaire respondents may limit the generalizability of the findings to other geographic regions or demographic groups, such as migrant workers. Future studies would benefit from employing stratified sampling methods across different Chinese cities and cultural contexts to ensure better representation of all demographic segments and enhance the external validity of the results. Second, cross-cultural comparative studies can inform the development of culturally tailored interventions to promote formal PBR. Research comparing recycling behaviors across different cultural contexts could illuminate how cultural factors, particularly along the individualism–collectivism spectrum, shape the psychological mechanisms underlying recycling intentions. Such investigations could inform the development of culturally tailored interventions. Third, the cross-sectional nature of the data may prevent definitive causal conclusions. Longitudinal or experimental designs tracking how these relationships evolve would strengthen causal claims about the influence of place attachment on recycling behaviors in response to changing policy environments.

Fourth, this theoretical framework focused specifically on establishing “reasons for” recycling through place attachment mechanisms, without examining the potential “reasons against” aspect of BRT, such as inconvenience, lack of infrastructure, or economic disincentives. Future research could enhance the BRT framework by investigating how these barriers moderate the relationship between intentions and actual recycling behaviors. Understanding both supportive and inhibiting factors in future research will be crucial for developing effective interventions and policies to promote recycling behavior. Fifth, no mediation analysis was used, and linear relationships between variables were assumed, which may not capture the full complexity of the underlying processes. While PA explains substantial variance in SA, other factors may play complementary roles. Future research could explore alternative statistical approaches to elucidate more nuanced mediation and moderation pathways. For example, research examining the effectiveness of economic incentives, environmental knowledge, specific policy interventions, and educational campaigns in conjunction with psychological factors would provide valuable insights for developing comprehensive recycling promotion strategies. Finally, technological solutions have the potential to streamline recycling processes and enhance transparency and accountability in the recycling supply chain. Further investigation is warranted into the role of technological innovations (Cheng et al., 2023), such as smart recycling systems and blockchain-based tracking mechanisms, in facilitating formal recycling.

## Data Availability

The original contributions presented in the study are included in the article/Supplementary material, further inquiries can be directed to the corresponding author.
