# Supplementary material for: Psychological drivers of electric vehicle battery recycling: the impact of place attachment and sustainable attitudes
Source: Front Psychol. 2026 Jan 22;16:1634913. doi: 10.3389/fpsyg.2025.1634913 (PMC12872936; doi:10.3389/fpsyg.2025.1634913)
Supplement: Supplementary file 1 [file Table_1.DOCX]

**Appendix 1.** Questionnaire

| **Variable** | **Item** | **Question** |
| --- | --- | --- |
| PI | PI1 | This city means a lot to me |
|  | PI2 | I am very attached to this city |
|  | PI3 | I identify strongly with this city |
|  | PI4 | I feel no commitment to this city |
| SB | SB1 | I have a lot of fond memories about this city |
|  | SB2 | I have a special connection to this city and the people |
|  | SB3 | I do tell many people about this city |
|  | SB4 | I would bring my friends or families to this city |
| NB | NB1 | I am very attached to the natural environment in the city |
|  | NB2 | When I spend time in the natural environment in the city, I feel at peace with myself |
|  | NB3 | I learn a lot about myself when spending time in the natural environment in the city |
|  | NB4 | When I spend time in the natural environment in the city, I feel a deep sense of oneness with the natural environment |
| ECA | ECA1 | Recycling leads to the reuse of power batteries for the production of raw materials and energy through recycling. |
|  | ECA2 | Recycling reduces the generation of waste. |
|  | ECA3 | Recycling leads to savings in resource consumption for the production of power batteries. |
|  | ECA4 | Recycling leads to saving money and using it for other projects. |
| SA  EA | SA1 | Recycling leads to a boom in job opportunities for people in the community, such as those in collection, retail, and recycling labor. |
|  | SA2 | Recycling leads to the promotion of a sustainable lifestyle. |
|  | SA3 | Recycling leads to the credibility of formal power batteries recycling organizations that preserve nature in the public perception. |
|  | ENA1 | Recycling prevents pollution of water, air, soil, rivers, or seas. |
| SA | ENA2 | Recycling prevents the creation of landfills and sewage or the degradation of natural beauty. |
|  | ENA3 | Recycling prevents public health problems. |
|  | ENA4 | Recycling saves space in landfills by reducing waste from power batteries. |
| INT | INT1 | I will adopt the online e-waste recycling platform in the future. |
|  | INT2 | My willingness to use the online e-waste recycling platform is high. |
|  | INT3 | I want to carry on using the online e-waste recycling platform. |
|  | INT4 | The possibility of me using the online e-waste recycling platform is high. |

**Appendix 2.** Reliability and convergent validity.

| **Construct** | **Item** | **Loading** | **CA** | **CR** | **AVE** |
| --- | --- | --- | --- | --- | --- |
| ECA | ECA1 | 0.865 | 0.863 | 0.866 | 0.709 |
|  | ECA2 | 0.812 |  |  |  |
|  | ECA3 | 0.866 |  |  |  |
|  | ECA4 | 0.824 |  |  |  |
| ENA | ENA1 | 0.872 | 0.901 | 0.903 | 0.771 |
|  | ENA2 | 0.896 |  |  |  |
|  | ENA3 | 0.886 |  |  |  |
|  | ENA4 | 0.857 |  |  |  |
| INT | INT1 | 0.890 | 0.858 | 0.869 | 0.700 |
|  | INT2 | 0.794 |  |  |  |
|  | INT3 | 0.855 |  |  |  |
|  | INT4 | 0.806 |  |  |  |
| NB | NB1 | 0.827 | 0.848 | 0.853 | 0.688 |
|  | NB2 | 0.786 |  |  |  |
|  | NB3 | 0.867 |  |  |  |
|  | NB4 | 0.835 |  |  |  |
| PI | PI1 | 0.773 | 0.776 | 0.789 | 0.599 |
|  | PI2 | 0.849 |  |  |  |
|  | PI3 | 0.709 |  |  |  |
|  | PI4 | 0.757 |  |  |  |
| SA | SA1 | 0.876 | 0.784 | 0.788 | 0.699 |
|  | SA2 | 0.829 |  |  |  |
|  | SA3 | 0.802 |  |  |  |
| SB | SB1 | 0.703 | 0.723 | 0.729 | 0.546 |
|  | SB2 | 0.816 |  |  |  |
|  | SB3 | 0.703 |  |  |  |
|  | SB4 | 0.728 |  |  |  |

**Appendix 3.** Cross-loadings

|  | **ECA** | **ENA** | **INT** | **NB** | **PI** | **SA** | **SB** |
| --- | --- | --- | --- | --- | --- | --- | --- |
| ECA1 | 0.865 | 0.531 | 0.510 | 0.481 | 0.541 | 0.377 | 0.505 |
| ECA2 | 0.812 | 0.424 | 0.478 | 0.482 | 0.403 | 0.313 | 0.449 |
| ECA3 | 0.866 | 0.465 | 0.508 | 0.500 | 0.496 | 0.397 | 0.467 |
| ECA4 | 0.824 | 0.456 | 0.547 | 0.551 | 0.556 | 0.476 | 0.516 |
| ENA1 | 0.456 | 0.872 | 0.456 | 0.398 | 0.369 | 0.375 | 0.410 |
| ENA2 | 0.489 | 0.896 | 0.450 | 0.494 | 0.377 | 0.350 | 0.423 |
| ENA3 | 0.473 | 0.886 | 0.491 | 0.466 | 0.430 | 0.377 | 0.489 |
| ENA4 | 0.543 | 0.857 | 0.442 | 0.413 | 0.420 | 0.340 | 0.434 |
| INT1 | 0.584 | 0.465 | 0.890 | 0.514 | 0.658 | 0.449 | 0.565 |
| INT2 | 0.436 | 0.415 | 0.794 | 0.416 | 0.507 | 0.353 | 0.488 |
| INT3 | 0.542 | 0.514 | 0.855 | 0.443 | 0.633 | 0.398 | 0.545 |
| INT4 | 0.456 | 0.344 | 0.806 | 0.431 | 0.462 | 0.373 | 0.486 |
| NB1 | 0.482 | 0.428 | 0.415 | 0.827 | 0.350 | 0.485 | 0.380 |
| NB2 | 0.447 | 0.425 | 0.430 | 0.786 | 0.296 | 0.339 | 0.359 |
| NB3 | 0.534 | 0.435 | 0.513 | 0.867 | 0.477 | 0.462 | 0.448 |
| NB4 | 0.521 | 0.394 | 0.434 | 0.835 | 0.427 | 0.511 | 0.359 |
| PI1 | 0.465 | 0.358 | 0.559 | 0.351 | 0.773 | 0.377 | 0.376 |
| PI2 | 0.526 | 0.440 | 0.540 | 0.431 | 0.849 | 0.428 | 0.466 |
| PI3 | 0.376 | 0.262 | 0.553 | 0.384 | 0.709 | 0.374 | 0.314 |
| PI4 | 0.466 | 0.328 | 0.476 | 0.291 | 0.757 | 0.364 | 0.389 |
| SA1 | 0.459 | 0.417 | 0.413 | 0.442 | 0.469 | 0.876 | 0.422 |
| SA2 | 0.402 | 0.344 | 0.349 | 0.398 | 0.384 | 0.829 | 0.386 |
| SA3 | 0.313 | 0.267 | 0.417 | 0.524 | 0.393 | 0.802 | 0.346 |
| SB1 | 0.352 | 0.280 | 0.498 | 0.265 | 0.344 | 0.318 | 0.703 |
| SB2 | 0.441 | 0.419 | 0.477 | 0.378 | 0.365 | 0.349 | 0.816 |
| SB3 | 0.492 | 0.453 | 0.474 | 0.410 | 0.425 | 0.360 | 0.703 |
| SB4 | 0.396 | 0.291 | 0.395 | 0.296 | 0.342 | 0.326 | 0.728 |

**Appendix 4.** Discriminant validity (Fornell–Larcker criterion).

|  | **ECA** | **ENA** | **INT** | **NB** | **PI** | **SA** | **SB** |
| --- | --- | --- | --- | --- | --- | --- | --- |
| ECA | 0.842 |  |  |  |  |  |  |
| ENA | 0.558 | 0.878 |  |  |  |  |  |
| INT | 0.608 | 0.524 | 0.837 |  |  |  |  |
| NB | 0.600 | 0.506 | 0.54 | 0.829 |  |  |  |
| PI | 0.597 | 0.455 | 0.683 | 0.471 | 0.774 |  |  |
| SA | 0.468 | 0.411 | 0.472 | 0.546 | 0.498 | 0.836 |  |
| SB | 0.577 | 0.501 | 0.625 | 0.467 | 0.504 | 0.461 | 0.739 |

**Appendix 5.** Discriminant validity (HTMT).

|  | **ECA** | **ENA** | **INT** | **NB** | **PI** | **SA** | **SB** |
| --- | --- | --- | --- | --- | --- | --- | --- |
| ECA |  |  |  |  |  |  |  |
| ENA | 0.632 |  |  |  |  |  |  |
| INT | 0.698 | 0.59 |  |  |  |  |  |
| NB | 0.697 | 0.579 | 0.631 |  |  |  |  |
| PI | 0.719 | 0.536 | 0.833 | 0.575 |  |  |  |
| SA | 0.564 | 0.488 | 0.570 | 0.662 | 0.637 |  |  |
| SB | 0.717 | 0.603 | 0.789 | 0.582 | 0.660 | 0.607 |  |

**Appendix 6.** Model fit.

|  | **Saturated model** | **Estimated model** |
| --- | --- | --- |
| SRMR | 0.062 | 0.077 |
| d_ULS | 1.435 | 2.263 |
| d_G | 0.536 | 0.618 |
| Chi-square | 1351.688 | 1465.140 |
| NFI | 0.919 | 0.902 |
